# Long-term survival outcomes of pediatric adrenal malignancies: An analysis with the upstaged SEER registry during 2000-2019

**DOI:** 10.3389/fendo.2022.977105

**Published:** 2022-09-12

**Authors:** Zemin Lv, Yunyun Yu, Yangmei Luo, Song Lin, Xuang Xiang, Xiaowen Mao, Shigang Cheng

**Affiliations:** ^1^ Department of Pediatric Surgery, Maternal and Child Health Hospital of Hubei Province, Tongji Medical College, Huazhong University of Science and Technology, Wuhan City, China; ^2^ Department of Health Science Center, Yangtze University, Jingzhou City, China

**Keywords:** child, adrenal malignancy, treatment, survival, prognosis

## Abstract

**Objective:**

To investigate the clinicopathological characteristics and long-term survival outcomes of pediatric adrenal malignancies.

**Method:**

This study retrospectively analyzed children with pathologically confirmed pediatric adrenal malignancies from Surveillance, Epidemiology, and End Results Database from 2000 to 2019. Kaplan-Meier curve was used to assess the overall survival (OS) and cancer-special survival (CSS), and the Log-Rank method was used to calculate statistical differences. Cox proportional hazards model and Fine-and-Grey model were used to calculate the hazard ratio (HR) of all-cause mortality risk and the sub-distribution HR (sHR) of disease-specific mortality risk, respectively, and their corresponding 95% confidence intervals (CI).

**Results:**

1601 children were included in the study in which 1335 (83.4%) neuroblastoma, 151 (9.4%) ganglioneuroblastoma, 89 (5.6%) adrenocortical carcinoma, and 26 (1.6%) were diagnosed with other types malignancies. Metastatic disease accounted for the largest proportion (69.3%), and the proportion of metastases diagnosed by neuroblastoma was higher than that of adrenocortical carcinoma and ganglioneuroblastoma (73.9% vs. 45.7% vs. 47.2%). The 5-year OS and CSS of all cohort were 69.5% and 70.5%, respectively. Adrenal cortical carcinoma had the worst prognosis, with 5-year OS and CSS of 52.5% and 53.1%, respectively. Patients in recent years had no better OS and CSS than in previous years at diagnosis. The tumor stage remained the main prognostic predictor. Compared to metastatic adrenal tumors, the risk of all-cause mortality (adjusted HR: 0.12, 95% CI: 0.06-0.25, *P* < 0.001) and the risk of disease-specific mortality (adjusted sHR: 0.11, 95% CI: 0.05-0.25, *P*<0.001) was significantly lower for patients with localized diseases. Additionally, higher age, adrenal cortical carcinoma, and lack of complete tumor resection are independent risk factors for poor prognosis. Furthermore, it was found that the prognosis of patients who received chemotherapy was worse than those who did not, mainly because the former mostly had metastasis at the presentation and complete resection of the tumor cannot be achieved.

**Conclusion:**

The clinicopathological characteristics of pediatric adrenal malignancies have not changed significantly in the past two decades, while the prognosis of patients has improved. Early diagnosis of disease and complete resection of local tumors are the keys to improving prognosis.

## Introduction

Pediatric malignancies of adrenal glands are rare but have become one of the leading causes of death in pediatrics (1, 2). Their onset is usually stealthy, discovered incidentally, or identified by recognizing symptoms associated with excess hormone secretion (3, 4). Neuroblastoma, ganglioneuroblastoma, and adrenal cortical cancer account for most pediatric adrenal malignancies. Adrenal cortical cancer rarely occurs and has a poor prognosis (5–8). Pediatric malignant patients have benefited from the rapid progress in tumor treatment as the optimization of multimodality therapy and early tumor screening has dramatically improved the survival rate of pediatric malignant tumors (9–13). Given the low incidence of pediatric adrenal tumors, the small sample size, and the short follow-up time of prior studies on pediatric adrenal malignancies, the clinicopathological characteristics, treatment status, and prognosis are not well understood (2, 4, 11–15). Here, we used the Surveillance, Epidemiology, and End Results (SEER) database to study pediatric malignancies of adrenal glands to explore the long-term follow-up survival outcomes and prognostic risk analysis of pediatric adrenal malignancies.

## Patients and methods

### Data source

All data were obtained from the SEER database of 18 registries (https://seer.cancer.gov/). Since SEER databases were anonymized and were not associated with human research, therefore, the need for ethics approval was waived by the Ethics Review Board of our institute. As a retrospective study, the patients diagnosed with primary adrenal malignancy from 2000 to 2019 were identified. Patients ≤19 years and having adrenal cortical carcinoma, adrenal ganglioneuroblastoma, adrenal neuroblastoma, and other non-common tumors were identified. All diagnoses were confirmed by histology and not by autopsy or death certification, and all patients had a detailed cause of death and follow-up data. Cases with missing values were excluded.

### Study variables

The study mainly included the following variables for data analysis: year of diagnosis (2000-2004, 2005-2009, 2010-2014, and 2015-2019), age at diagnosis (0-4 years, 5-9 years, 10-14 year and 15+ year), gender (male and female), race (white, black and other), median household income ($0-$59999, $60000-$69999 and $70000+), residence locality (metropolitan and non-metropolitan), tumor size, tumor stage (distant, localized and regional), metastatic site (bone, brain, liver, lung), surgery and approaches (none, local tumor destruction/excision, radical surgery with or without other organs and simple/partial surgical removal), metastatic surgery Treatment (yes or no), chemoradiation (yes or no).

### Statistical analysis

The continuous variables were described as mean ± standard deviation (SD) for data with a normal distribution and compared with the student t-test. For non-normal distribution, the Wilcoxon rank-sum test was used to compare the data, and the data were described as median and interquartile range (IQR). Classification variables were represented by frequency (%) and compared by the chi-square test. OS and CSS were the primary endpoints of interest for this study. Kaplan-Meier was used to calculate OS and CSS. The hazard ratio (HR), the Sub-distribution hazard ratio (sHR), and the corresponding 95% confidence Interval (95% CI) of all-cause death and adrenal tumor-specific death were calculated by Cox proportional hazards model and Fine and Grey model, respectively. Logistic regression analysis was done to calculate odds ratios (OR) and their corresponding 95% CI for factors associated with treatment choice. All analyses were conducted using R (Version 42.0). *P* < 0.05 (two-sided) was considered statistically significant.

## Results

### Baseline characteristics

**Figure 1:**
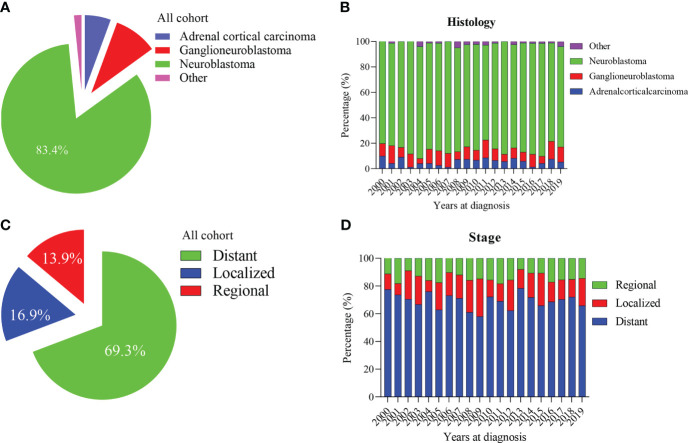
Percentage of pediatric adrenal malignancies by histology and tumor stage. **(A, B)** Percentage of histology in all cohorts and stratified by year at diagnosis. **(C, D)** Percentage of tumor stage in all cohorts and stratified by year at diagnosis.

Our study included 1601 patients with adrenal-derived malignant tumors. The primary histological type was neuroblastoma (1335 cases, 83.4%). Ganglioneuroblastoma and adrenal cortical carcinoma were the other two main pathological types, accounting for 9.4% and 5.6%, respectively, whereas other rare cancers accounted for only 1.6% **(** **Table 1** and **Figure 1A** **)**. The incidence of neuroblastoma did not change much from 2000 to 2019 **(** **Figure 1B** **)**. Most cases had an onset in the age group of 0-4 years, accounting for 79.9% of the cases. Neuroblastoma accounted for the largest proportion (86.4%) in 0-4 years old. However, ganglioneuroblastoma and adrenal cortical carcinoma accounted for 55.6% and 41.5%, respectively; furthermore, the proportion of other rare cases was 19.2% in the older age groups (**Table 1**).

**Table 1:** Baseline characteristics of the study cohort.

		By histology			
	ALL N=1601	Neuroblastoma N=l335 (83.4%)	Ganglioneuroblastoma N=l51 (9.4%)	Adrenal cortical carcinoma N=89 5.6%	Other N=26 (1.6%)
**Year at diagnosis**	** **			** **	
2000-2004	374 (23.4%)	315 (23.6%)	34 (22.5%)	21 (23.6%)	4 (15.4%)
2005-2009	421 (26.3%)	352 (26.4%)	42 (27.8%)	19 (21.3%)	8 (30.8%)
2010-2014	411 (25.7%)	339 (25.4%)	36 (23.8%)	29 (32.6%)	7 (26.9%)
2015-2019	395 (24.7%)	329 (24.6%)	39 (25.8%)	20 (22.5%)	7 (26.9%)
**Age at diagnosis. Median (IQR)**	2.00 [0.00;4.00]	1.00 [0.00;3.00]	4.00 [2.00;7.00]	9.00 [2.00;15.0]	15.0 [8.25;17.0]
**Age at diagnosis**					
0-4 year	1279 (79.9%)	1153 (86.4%)	84 (55.6%)	37 (41.6%)	5 (19.2%)
5-9 year	190 (11.9%)	136 (10.2%)	41 (27.2%)	9 (10.1%)	4 (15.4%)
10-14 year	73 (4.56%)	32 (2.40%)	18 (11.9%)	20 (22.5%)	3 (11.5%)
15+ year	59 (3.69%)	14 (1.05%)	8 (5.30%)	23 (25.8%)	14 (53.8%)
**Sex**					
Female	722 (45.1%)	580 (43.4%)	77 (51.0%)	53 (59.6%)	12 (46.2%)
Male	879 (54.9%)	755 (56.6%)	74 (49.0%)	36 (40.4%)	14 (53.8%)
**Race**					
White	1234 (77.1%)	1026 (76.9%)	108 (71.5%)	78 (87.6%)	22 (84.6%)
Black	214 (13.4%)	179 (13.4%)	27 (17.9%)	6 (6.74%)	2 (7.69%)
Other	153 (9.56%)	130 (9.74%)	16 (10.6%)	5 (5.62%)	2 (7.69%)
**Median household income**					
$0-$59999	406 (25.4%)	335 (25.1%)	44 (29.1%)	22 (24.7%)	5 (19.2%)
$60000-$69999	498 (31.1%)	412 (30.9%)	49 (32.5%)	28 (31.5%)	9 (34.6%)
$70000+	697 (43.5%)	588 (44.0%)	58 (38.4%)	39 (43.8%)	12 (46.2%)
**Residence**					
Metropolitan	1448 (90.4%)	1214 (90.9%)	131 (86.8%)	79 (88.8%)	24 (92.3%)
Nonmetropolitan	153 (9.56%)	121 (9.06%)	20 (13.2%)	10 (11.2%)	2 (7.69%)
**Size. Median (IQR)**	73.0 [50.0;105]	75.0 [49.0; 105]	61.0 [43.5;84.5]	102 [70.8;134]	70.0 [50.5;102]
**Stage**					
Distant	1109 (69.3%)	986 (73.9%)	69 (45.7%)	42 (47.2%)	12 (46.2%)
Localized	270 (16.9%)	167 (12.5%)	58 (38.4%)	34 (38.2%)	11 (42.3%)
Regional	222 (13.9%)	182 (13.6%)	24 (15.9%)	13 (14.6%)	3 (11.5%)
Bone metastasis *					
Bone	375 (47.5%)	342 (52.5%)	22 (29.3%)	6 (12.5%)	5 (35.7%)
No	414 (52.5%)	310 (47.5%)	53 (70.7%)	42 (87.5%)	9 (64.3%)
**Brain metastasis** *					
Brain	44 (5.6%)	42 (6.4%)	2 (2.7%)	0 (0%)	0 (0%)
No	745 (94.4%)	610 (93.6%)	73 (97.3%)	48 (100%)	14 (100%)
**Liver metastasis** *					
Liver	169 (21.4%)	148 (22.7%)	3 (4.0%)	16 (33.3%)	2 (14.3%)
No	620 (78.6%)	504 (77.3%)	72 (96.0%)	32 (66.7%)	12 (85.7%)
**Lung metastasis** *					
Lung	70 (8.9%)	48 (7.4%)	3 (4.0%)	13 (27.1%)	6 (42.9%)
No	719 (91.1%)	604 (92.6%)	72 (96.0%)	35 (72.9%)	8 (57.1%)
**Metastasis site** *					
No	318 (40.3%)	236 (36.2%)	51 (68.0%)	25 (52.1%)	6 (42.9%)
2+ sites	153 (19.4%)	133 (20.4%)	5 (6.7%)	11 (22.9%)	4 (28.6%)
Bone	234 (29.7%)	214 (32.8%)	17 (22.7%)	1 (2.1%)	2 (14.3%)
Liver	72 (9.1%)	65 (10.0%)	1 (13%)	6 (12.5%)	0 (0%)
Lung	12 (15%)	4 (0.6%)	1 (13%)	5 (10.4%)	2 (14.3%)
**Surgical treatment**					
No	292 (18.2%)	266 (19.9%)	5 (3.31%)	15 (16.9%)	6 (23.1%)
Local tumor destruction/excision	139 (8.68%)	113 (8.46%)	16 (10.6%)	9 (10.1%)	1 (3.85%)
Radical surgery with or without other organs	881 (55.0%)	728 (54.5%)	96 (63.6%)	46 (517%)	11 (42.3%)
Simple/partial surgical removal	289 (18.1%)	228 (17.1%)	34 (22.5%)	19 (213%)	8 (30.8%)
**Surgery for non-primary other distant sites**					
None	1393 (87.0%)	1157 (86.7%)	135 (89.4%)	79 (88.8%)	22 (84.6%)
Yes	208 (13.0%)	178 (13.3%)	16 (10.6%)	10 (112%)	4 (15.4%)
**Radiotherapy**					
No	1104 (69.0%)	885 (66.3%)	116 (76.8%)	81 (910%)	22 (84.6%)
Yes	497 (310%)	450 (33.7%)	35 (23.2%)	8 (8.99%)	4 (15.4%)
**Chemotherapy**					
No/Unknown	358 (22.4%)	235 (17.6%)	70 (46.4%)	37 (416%)	16 (615%)
Yes	1243 (77.6%)	1100 (82.4%)	81 (53.6%)	52 (58.4%)	10 (38.5%)
**Months to treatment**					
0 months	1222 (76.3%)	1012 (75.8%)	115 (76.2%)	76 (85.4%)	19 (73.1%)
1 months	331 (20.7%)	281 (210%)	33 (219%)	12 (13.5%)	5 (19.2%)
2+ months	48 (3.00%)	42 (3.15%)	3 (1.99%)	1 (1.12%)	2 (7.69%)

IQR, Interquartile range.

*Data with complete information of metastasis organ.

**Figure 2:**
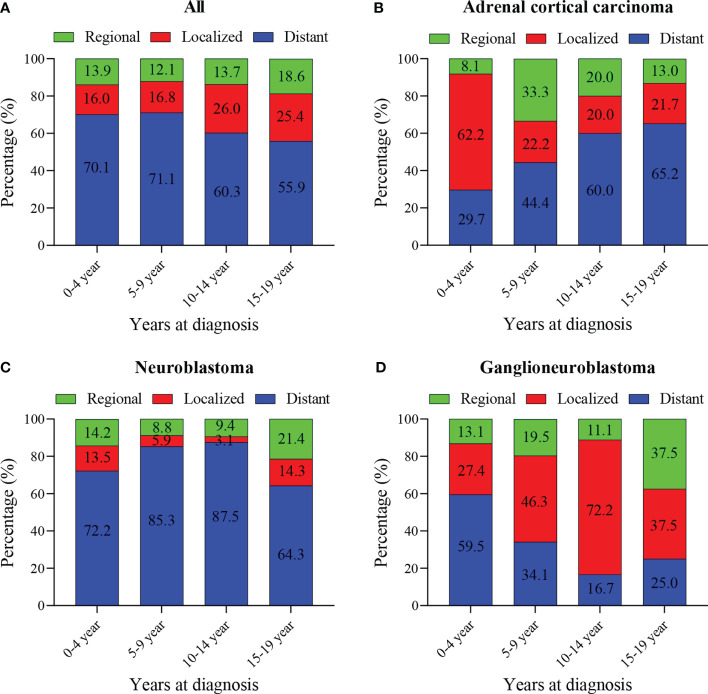
Percentage of pediatric adrenal malignancies by age at diagnosis, tumor stage and histology. **(A)** Percentage of stage in all cohorts and stratified by age at diagnosis. **(B)** Percentage of stage in Adrenal cortical carcinoma cohorts and stratified by age at diagnosis. **(C)** Percentage of stage in neuroblastoma cohorts and stratified by age at diagnosis. **(D)** Percentage of stage in ganglioneuroblastoma cohorts and stratified by age at diagnosis.

The median tumor size (102mm) of adrenal cortical carcinoma was larger than other tumor types (neuroblastoma- 75mm, ganglioneuroblastoma- 61mm and others- 70mm). Most of the children had metastatic diseases at the time of diagnosis (**Figure 1C**). This condition has not changed much in these two decades (**Figure 1D**). Patients with neuroblastoma present diststant metastasis accounted for 73.9%, while Ganglioneuroblastoma and adrenal cortical carcinoma accounted for 45.7% and 47.2%, respectively. Bone was the most common metastasis site (about 80.0% of all metastasis cases, data did not present here). **Figure 2A** shows that about 70% of children under 9 years old had disease metastasis at the time of treatment (0 – 4 years old: 70.1%, 5 – 9 years old: 71.1%); although the proportion of children over 10 years old with metastasis decreased, it still more than the general children with disease metastasis (10-14 years old: 60.3%; 15-19 years old: 55.9%). However, we found that with the increase in age, the proportion of adrenal cortical carcinoma patients with metastasis increased (**Figure 2B**). The proportion of metastasis from adrenal neuroblastoma is higher in all age stages. In contrast, the proportion of metastatic lesions in Ganglioneuroblastoma is lower (**Figures 2C, D** **)**.

Most children received surgical treatment (8.68% local tumor destruction/excision, 55.0% radical surgery with or without other organs, and 18.1% simple/partial surgical removal), and a few did not (18.2%). At the same time, 77.6% and 31.0% of patients received chemotherapy and radiotherapy, respectively. Additionally, 13.0% of patients also received surgical treatment for distant lymph node or organ metastasis, and most (76.3%) patients underwent either surgery or radiotherapy and chemotherapy within 1 month after diagnosis (**Table 1**).

### Survival outcomes

**Figure 3:**
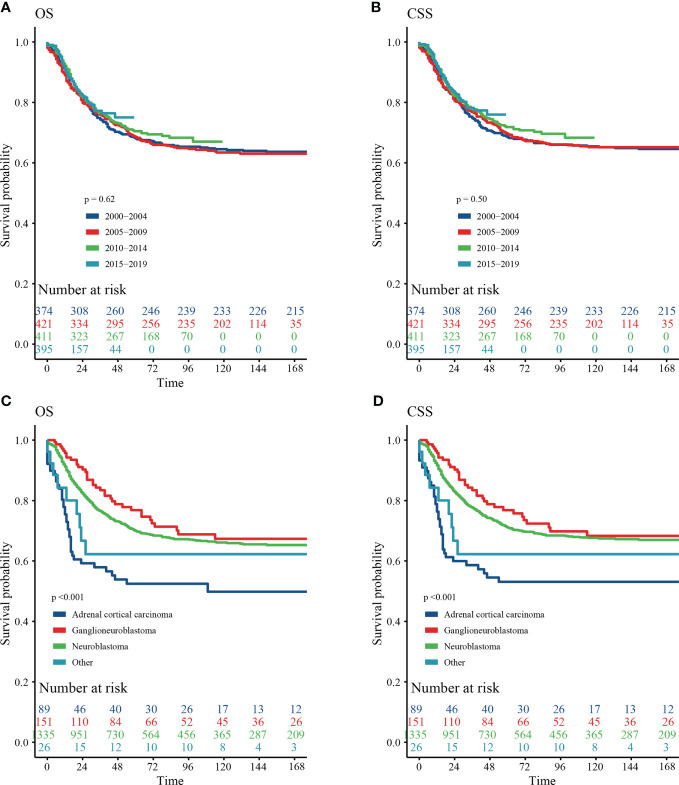
Survival curve of analyzed by Kaplan–Meier method. **(A)** Overall survival (OS) stratified by year at diagnosis. **(B)** Cancer-special survival (CSS) stratified by year at diagnosis. **(C)** OS stratified by histology. **(D)** CSS stratified by histology.

**Figure 4:**
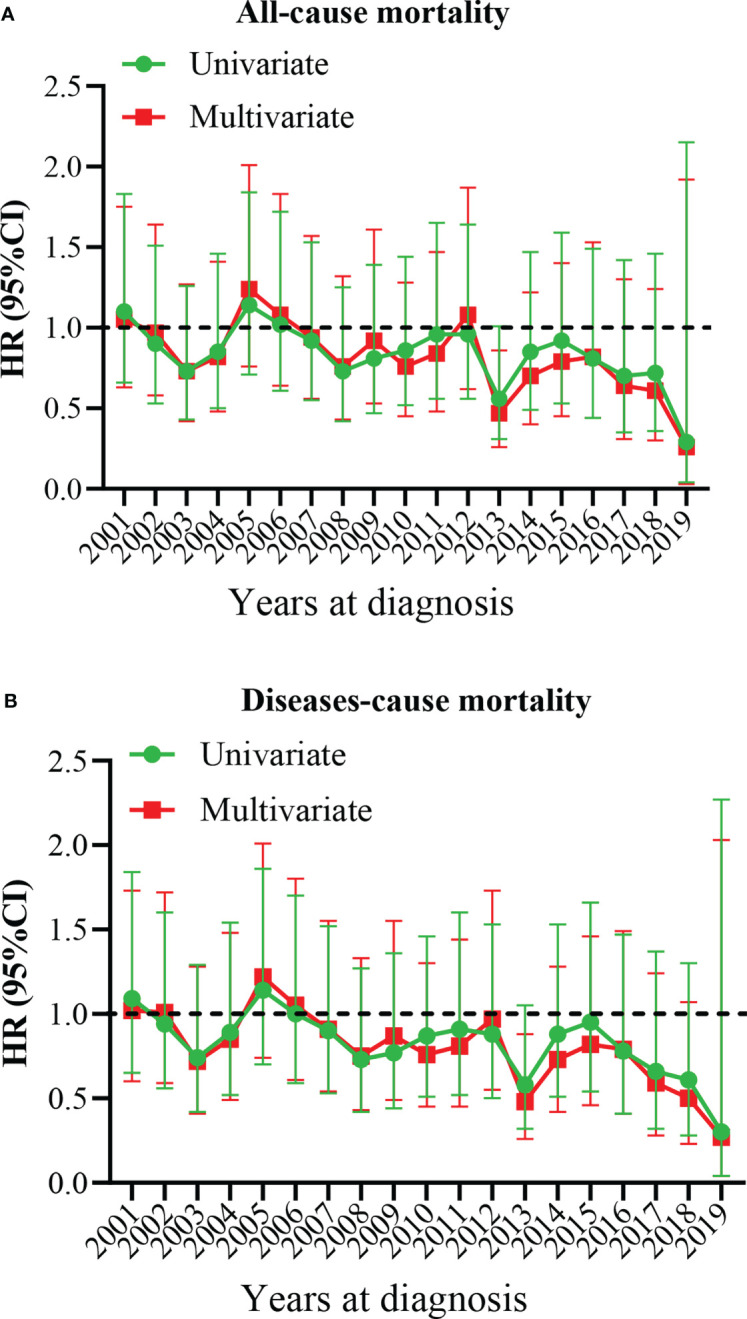
Multivariate hazard ratio (HR) analysis for all-cause mortality and diseases-cause mortality. **(A)** All-cause mortality. **(B)** Diseases-cause mortality. The year 2000 at diagnosis was considered as a reference.

Our results showed that OS and CSS didn’t change over the years (200-2019) (**Figures 3A, B** **)**. The OS and CSS of neuroblastoma and ganglioneuroblastoma was better than adrenal cortical carcinoma (**Figures 3C, D** **)**. The 5-year OS of the total population was 69.5% (95% CI: 67.1% – 72.0%, **Table 2**). The 5-year OS of metastatic patients was 59.3%, while that of local tumor patients was 89.3%. (**Table 2**). **Table 3** summarizes the risk factors of disease-specific death in 5-year CSS of different subgroups. Our results show that the overall 5-year CSS was 70.5% (95% CI: 68.1% -73.0%). The multivariate COX model showed that the age, histological type, tumor size, tumor stage, and treatment were independent risk factors for both, all-cause mortality and disease-specific death. Although in the recent year of the diagnosis had a lower risk of mortality compared to the prior years, the year of diagnosis was neither a significant risk predictor of all-cause mortality (**Figure 4A**) nor disease-cause mortality (**Figure 4B**).

**Table 2:** Five-year overall survival and predictors of all-cause mortality.

	5-year overall survival		Univariable#			Multivariable#	
	rate (95%CI)		HR (95%CI)	P		HR (95%CI)	P
**All cohort**	69.5% [67.1%-72.0%]						
**Year at diagnosis**							
2000-2004	68.4% [63.8%-73.2%]		1 reference			1 reference	
2005-2009	68.8% [64.4%-73.4%]		1.02 [0.81-1.29]	0.856		110 [0.86-1.40]	0.4578
2010-2014	70.5% [66.1%-75.2%]		0.91 [0.71-1.16]	0.454		0.82 [0.63-106]	0.1317
2015-2019*	75 0% [69.2%-81.4%]		0.86 [0.64-1.17]	0.35		0.79 [0.58-110]	0.1623
**Age at diagnosis**							
0-4 year	72.8% [70.2%-75.4%]		1 reference			1 reference	
5-9 year	60.7% [53.4%-69.0%]		1.58 [1.22-2.03]	<0.001		1.56 [1.20-2.03]	0.001
10-14 year	53 0% [41.7%-67.4%]		2.18 [1.53-3.10]	<0.001		2.15 [146-3.17]	<0.001
15+ year	42.9% [310%-59.4%]		2.87 [2.00-4.13]	<0.001		2.54 [155-4.17]	<0.001
**Sex**							
Female	69.3% [65.7%-73.1%]		1 reference			1 reference	
Male	69.6% [66.4%-73.0%]		1.03 [0.86-1.24]	0.751		1.02 [0.84-1.23]	0.8728
**Race**							
White	71.7% [69.0%-74.4%]		1 reference			1 reference	
Black	62.5% [55.9%-70.0%]		1.37 [107-1.75]	0.013		1.28 [0.99-166]	0.0631
Other	619% [54.1%-70.8%]		1.40 [1.05-1.88]	0.023		1.31 [0.96-1.79]	0.0872
**Median household income**							
$0-$59999	64.8% [59.9%-70.0%]		1 reference			1 reference	
$60000-$69999	69.3% [65.1%-73.9%]		0.85 [0.68-108]	0.18		0.91 [0.70-1.19]	0.4937
$70000+	72.5% [68.9%-76.2%]		0.77 [0.62-0.96]	0.021		0.80 [0.62-1.04]	0.0907
**Residence**							
Metropolitan	70.4% [67.9%-73.0%]		1 reference			1 reference	
Nonmetropolitan	60.2% [52.2%-69.3%]		1.39 [1.05-1.84]	0.019		1.26 [0.91-1.74]	0.1712
**Histology**							
Adrenal cortical carcinoma	52.5% [42.6%-64.6%]		1 reference			1 reference	
Ganglioneuroblastoma	76.8% [69.6%-84.8%]		0.43 [0.28-0.68]	<0.001		0.38 [0.23-0.63]	0.0002
Neuroblastoma	69.9% [67.3%-72.7%]		0.51 [0.37-0.71]	<0.001		0.32 [0.21-0.49]	<0.001
Other	62.3% [45.4%-85.3%]		0.79 [0.39-157]	0.497		0.72 [0.33-1.59]	0.4207
**Size. per cm**			1.05 [1.03-1.07]	<0.001		1.03 [1.02-1.05]	<0.001
**Stage**							
Distant	59.3% [56.2%-62.5%]		1 reference			1 reference	
Localized	96.1% [93.6%-98.6%]		0.09 [0.05-0.16]	<0.001		0.12 [0.06-0.25]	<0.001
Regional	89.3% [85.0%-93.9%]		0.23 [0.15-0.34]	<0.001		0.29 [0.19-0.45]	<0.001
**Surgical treatment**							
No	60.9% [55.3%-67.2%]		1 reference			1 reference	
Local tumor destruction/excision	77.6% [70.5%-85.5%]		0.46 [0.31-0.69]	<0.001		0.72 [0.46-1.11]	0.1388
Radical surgery with or not other organs	69.2% [66.0%-72.6%]		0.69 [0.55-0.87]	<0.001		0.83 [0.64-1.07]	0.152
Simple/partial surgical removal	75.2% [69.9%-80.8%]		0.52 [0.38-0.70]	<0.001		0.70 [0.50-0.98]	0.0385
**Surgery for non-primary other distant sites**							
None	71.1% [68.5%-73.7%]		1 reference			1 reference	
Yes	58.9% [52.1%-66.6%]		1.49 [1.17-1.89]	<0.001		1.11 [0.86-1.43]	0.4085
**Radiotherapy**							
No	73.8% [71.1%-76.7%]		1 reference			1 reference	
Yes	59.8% [55.2%-64.7%]		1.47 [1.22-1.77]	<0.001		1.05 [0.84-1.30]	0.6871
**Chemotherapy**							
No/Unknown	93.2% [90.4%-96.0%]		1 reference			1 reference	
Yes	62.7% [59.9%-65.7%]		5.49 [3.75-8.05]	<0.001		2.68 [1.62-4.46]	<0.001
**Months to treatment**							
0 months	70.5% [67.8%-73.3%]		1 reference			1 reference	
1 months	64.2% [58.8%-70.1%]		1.26 [1.01-1.56]	0.037		0.98 [0.78-1.22]	0.8273
2+ months	78.9% [67.4%-92.3%]		0.84 [0.47-1.50]	0.565		1.12 [0.61-2.06]	0.72

HR, hazard ratio; CI, confidence interval;

#Cox proportional risk regression model;

*59 months survival rate.

**Table 3:** Five-year cancer-sQecial survival and Qredictors of mortality by adrenal cancer.

			Univariable#			Multivariable#	
	rate (95%CI)		sHR (95%CI)	P		sHR (95%CI)	P
**All cohort**	70.5% [68.1%-73.0%]						
**Year at diagnosis**							
2000-2004	68.8% [64.2%-73.6%]		1 reference			1 reference	
2005-2009	69.8% [65.5%-74.4%]		0.99 [0.78-1.25]	0.914		1.10 [0.82-1.46]	0.5229
2010-2014	71.8% [67.5%-76.5%]		0.88 [0.68-1.13]	0.32		0.92 [0.68-1.25]	0.5943
2015-2019*	76.0% [70.1%-82.3%]		0.82 [0.60-1.12]	0.215		0.83 [0.58-1.20]	0.3286
**Age at diagnosis**							
0-4 year	73.8% [71.3%-76.5%]		1 reference			1 reference	
5-9 year	61.6% [54.3%-70.0%]		1.59 [1.23-2.06]	<0.001		1.55 [1.16-2.09]	0.0034
10-14 year	53.0% [41.7%-67.4%]		2.29 [1.61-3.27]	<0.001		1.84 [0.24-2.39]	0.0025
15+ year	43.7% [31.6%-60.4%]		2.64 [1.79-3.89]	<0.001		1.85 [1.05-3.28]	0.0339
**Sex**							
Female	70.4% [66.9%-74.2%]		1 reference			1 reference	
Male	70.5% [67.3%-73.8%]		1.03 [0.86-1.25]	0.722		0.96 [0.77-1.19]	0.7076
**Race**							
White	72.3% [69.6%-75.0%]		1 reference			1 reference	
Black	64.4% [57.8%-71.9%]		1.33 [1.03-1.72]	0.027		1.25 [0.93-1.68]	0.1452
Other	64.7% [56.9%-73.6%]		1.32 [0.97-1.79]	0.077		1.33 [0.97-1.81]	0.0756
**Median household income**							
$0-$59999	66.3% [61.5%-71.5%]		1 reference			1 reference	
$60000-$69999	70.2% [66.0%-74.8%]		0.87 [0.68-1.10]	0.247		1.10 [0.82-1.48]	0.5397
$70000+	73.2% [69.7%-76.9%]		0.78 [0.62-0.98]	0.03		0.90 [0.67-1.22]	0.5113
**Residence**							
Metropolitan	71.4% [68.9%-74.0%]		1 reference	0.024		1 reference	0.1407
Nonmetropolitan	61.2% [53.3%-70.4%]		1.39 [1.04-1.84]			1.28 [0.92-1.77]	
**Histology**							
Adrenal cortical carcinoma	53.1% [43.2%-65.3%]		1 reference			1 reference	
Ganglioneuroblastoma	76.8% [69.6%-84.8%]		0.44 [0.28-0.70]	<0.001		0.44 [0.26-0.73]	0.0014
Neuroblastoma	71.1% [68.5%-73.8%]		0.51 [0.37-0.72]	<0.001		0.35 [0.23-0.53]	<0.001
Other	62.3% [45.4%-85.3%]		0.75 [0.36-1.55]	0.432		0.75 [0.29-1.89]	0.5364
**Size. per cm**			1.05 [1.03-1.07]	<0.001		1.04 [1.02-1.06]	<0.001
**Stage**							
Distant	60.5% [57.5%-63.8%]		1 reference			1 reference	
Localized	96.5% [94.2%-98.9%]		0.08 [0.04-0.15]	<0.001		0.11 [0.05-0.25]	<0.001
Regional	89.3% [85.0%-93.9%]		0.23 [0.15-0.35]	<0.001		0.28 [0.18-0.45]	<0.001
**Surgical treatment**							
No	62.6% [57.0%-68.9%]		1 reference			1 reference	
Local tumor destruction/excision	77.6% [70.5%-85.5%]		0.47 [0.31-0.71]	<0.001		0.67 [0.41-1.10]	0.1144
Radical surgery with or not other organs	70.2% [67.0%-73.6%]		0.69 [0.55-0.87]	0.002		0.80 [0.59-1.09]	0.1531
Simple/partial surgical removal	76.0% [70.7%-81.6%]		0.53 [0.39-0.72]	<0.001		0.63 [0.42-0.94]	0.0253
**Surgery for non-primary other distant sites**							
None	72.0% [69.5%-74.6%]		1 reference			1 reference	
Yes	60.3% [53.4%-68.1%]		1.49 [1.16-1.90]	0.002		1.13 [0.85-1.51]	0.3915
**Radiotherapy**							
No	74.9% [72.2%-77.7%]		1 reference			1 reference	
Yes	60.7% [56.1%-65.6%]		1.47 [1.21-1.77]	<0.001		0.95 [0.75-1.22]	0.7048
**Chemotherapy**							
No/Unknown	94.0% [91.4%-96.7%]		1 reference			1 reference	
Yes	63.8% [60.9%-66.8%]		6.69 [4.36-10.27]	<0.001		2.61 [1.47-4.64]	0.001
**Months to treatment**							
0 months	71.3% [68.6%-74.1%]		1 reference			1 reference	
1 months	66.0% [60.6%-71.9%]		1.22 [0.98-1.52]	0.081		0.92 [0.71-1.19]	0.5188
2+ months	78.9% [67.4%-92.3%]		0.81 [0.44-1.48]	0.491		1.40 [0.68-2.87]	0.3594

sHR, Sub-distribution hazard ratio; CI, confidence interval;

#Fine and Grey regression model;

*59 months survival rate.

It’s puzzling that the survival analysis showed that chemotherapy did not benefit patients but increased the risk of death. However, it should be noted that chemotherapy was significantly correlated with the recent year of diagnosis, younger age of patients at diagnosis, metropolitan patient’s, larger tumor size, tumor with distant disease, nonsurgical treatment performed (**Supplementary Table 1**).

Considering the differences in treatment and prognosis between adrenocortical carcinoma and adrenal neuroblastoma/ganglioneuroblastoma, we will perform survival analysis for the two subgroups separately. We found that OS in the adrenal neuroblastoma/ganglioneuroblastoma subgroups correlated with patient age (the older the age, the worse the OS), race (worse in blacks than whites), and tumor stage (worse in metastatic cases than in patients without metastases) (**Supplementary Table 2**). However, only tumor stage and complete tumor resection were independent prognostic factors for adrenocortical carcinoma (**Supplementary Table 3**).

## Discussion

Neuroblastoma is the most common pathological type of adrenal malignancy in children (7, 16), and approximately 46% of neuroblastomas arise from the adrenal gland (17). The primary site of the adrenal neuroblastoma is often concealed and extensive, but its biological behavior shows high malignancy and rapid growth and is prone to multiple early metases (18). Our study found that ganglioneuroblastoma and neuroblastoma accounted for more than 80% of metastatic lesions. Since the clinical symptoms and signs of pediatric neuroblastoma are not specific, they are hard to diagnose. Hence, diagnosis is often missed, delaying treatment and losing the best time for the treatment. Therefore, delayed diagnosis is a major factor for the poor prognosis of neuroblastoma. With the improvement of multidisciplinary comprehensive treatment, the survival rate of patients has improved to a certain extent. However, our findings found that there was no statistically significant reduction in the risk of death in patients diagnosed recently compared with patients with pediatric adrenal malignancies diagnosed in the past.

Neuroblastoma is an embryonal tumor originating from primitive neural crest cells of the sympathetic nervous system. It affects the normal development of the adrenal medulla and paravertebral sympathetic ganglia, although its exact pathogenesis is still unclear (19). *MYCN* amplification is associated with the development of neuroblastoma. Numerous studies have shown that *MYCN* amplification correlates with neuroblastoma progressing rapidly, leading to a worse prognosis (20, 21). Therefore, drugs inhibiting *MYCN* amplification, such as retinoic acid, are used for maintenance therapy in high-risk children (22, 23).

Furthermore, the 1p, 11q loss, and 17q gain are also common chromosomal abnormalities in neuroblastoma (24–26). 11q aberration is reported in 20 to 45% of neuroblastoma, and 1p and 11q loss can render neuroblastomas in an undifferentiated or poorly differentiated state, increasing the risk of recurrence and leading to a poor prognosis (21, 25, 27, 28). Most children with neuroblastoma develop a 17q gain (80.4%), which, together with other unfavorable prognostic factors, contributes to the poor prognosis of neuroblastoma (21, 24).

Neuroblastoma may show biochemical abnormalities in blood and urine. Neuron-specific enolase (NSE) is an acid protease unique to neurons and neuroendocrine cells and a specific marker of neuroendocrine tumors (29, 30). NSE is expressed in neurons and is highly sensitive and specific to neuroblastoma and ganglioneuroblastoma. Its elevation often indicates advanced disease progression and poor prognosis. Studies have shown that NSE has a high positive expression rate in undifferentiated neuroblastoma and has high sensitivity and accuracy in monitoring tumor recurrence, especially in metastatic tumors. Urinary vanillylmandelic acid (VMA) is also an important indicator of early diagnosis of neuroblastoma (30, 31). A vast majority of neuroblastomas can also be accompanied by abnormal metabolism of catecholamines in the body. It can directly secrete the precursor substances of VMA, increasing its concentration in the blood and the urine. Lactate dehydrogenase (LDH) is an essential enzyme in the glycolytic pathway. The tumor tissue has the characteristics of high metabolism, and the serum LDH level can be used as an important indicator representing the tumor cell burden in the whole body, which is of great significance for the prognosis of neuroblastoma (29, 32, 33).

Surgically resecting the whole tumor tissue is the key to treating pediatric adrenal tumors (17). Our study showed that achieving complete tumor resection improves patient outcomes. However, when complete tumor resection cannot be performed, local surgery did not lead to better outcomes than patients without surgery. Without adjuvant radiotherapy and chemotherapy, children with localized diseases can achieve long-term survival after complete tumor resection and no surgical complications alone. However, in the diagnosis of pediatric adrenal malignancies, the tumor is often large, adheres tightly to the surrounding tissues, and even surrounds important blood vessels such as the inferior vena cava, making it difficult to operate on the tumor and remove it altogether. Blind pursuit of complete resection may also lead to more severe complications and affect the patient’s survival.

Patients with intermediate-risk tumors may receive chemotherapy to shrink the tumor before surgical removal (17, 34). Preoperative chemotherapy can significantly shrink the tumor, thicken the capsule, reduce the clinical stage of the tumor, and inhibit distant metastasis. The treatment model of neoadjuvant chemotherapy, surgical resection, adjuvant high-dose chemotherapy with hematopoietic stem cell rescue, and radiation therapy has become a classic mode of treatment for high-risk neuroblastoma (17, 35, 36). Chemotherapy can easily aggravate the degree of adhesion between the tumor and surrounding tissues and increase the difficulty of surgical resection. Moreover, chemotherapy can confuse or change the pathological staging, and special attention should be paid to the timing of surgery and chemotherapy. Prior studies have also found that surgical resection is vital in predicting outcomes for high-risk neuroblastomas that do not show a clinical response to induction treatment. However, gross total resection versus subtotal resection did not affect these outcomes (37).

Neuroblastoma is more sensitive to chemotherapy, but at the same time, it is necessary to pay attention to the toxic side effects of chemotherapy on children. Although high-intensity chemotherapy is used only for high-risk groups of neuroblastomata, the toxic side effects on children are significant and need vigilant analysis (38). An international, randomized, multi-arm, open-label, phase 3 trial found that Busulfan and melphalan improved event-free survival in children with high-risk neuroblastoma with an adequate response to induction treatment and caused fewer severe adverse events than carboplatin, etoposide, and melphalan treatment (38). A reasonable grasp of the intensity of chemotherapy is critical to avoid the effects of insufficient intensity in high-risk children and excessive intensity in low-risk children on prognosis.

In addition to surgical resection, radiotherapy can be a crucial treatment for neuroblastoma tumors (34). Local radiotherapy has an inhibitory effect on the recurrence of neuroblastoma. For high-risk patients, radiotherapy for primary and metastatic lesions can control the patient’s condition. Also, Iodine-131 metaiodobenzylguanidine (^131^I-MIBG) and radiolabeled DOTA-conjugated peptides have been reported to be useful for neuroblastoma treatment (39, 40). Because neuroblastoma cells highly express ganglioglyceride GD2 antibodies, they can be used as a better target for immunotherapy. Immunotherapy with an anti-GD2 antibody with GM-CSF, interleukin-2, and isotretinoin was associated with a significantly improved outcome compared with standard therapy in patients with high-risk neuroblastoma (41).

Adrenal cortical carcinoma in children is a rare adrenal malignancy, and its incidence is lower than that of neuroblastoma and ganglioneuroblastoma (10). In our study, adrenal cortical carcinoma patients only accounted for 5.6% of total patients. However, the degree of malignancy was very high in adrenal cortical carcinoma; the disease developed rapidly and had an infiltrative growth. The diagnosis is that the tumor volume is large and more likely to invade surrounding organs and tissues, making them hard to operate on (42). However, our study found that metastases in adrenocortical carcinoma are less common at the time of diagnosis than in neuroblastoma. The pathogenesis is unclear and can be associated with epigenetic alterations, manifesting as germline TP53 mutations or chromosome 11p abnormalities (8, 43). Compared to adult adrenal cortical carcinoma, pediatric adrenal cortical carcinoma has distinct features. It is closely associated with germline TP53 mutations, which are present in 53% of cases but only in < 10% in adults’ adrenal cortical carcinoma (44). At the time of onset, there may be indicators of excessive secretion of adrenal corticosteroids, such as hypertension, precocious puberty in childhood (manifested as male feminization, female virilization, penis enlargement, pubic hair development, deepening of the voice, breast development, etc.) and Cushing syndrome (manifested as a full moon face, bloody appearance, central obesity, acne, purple striae, hypertension, secondary diabetes, and osteoporosis, etc.). For pediatric adrenocortical carcinoma, 90% of patients presented with evidence of hormonal hypersecretion, and there was an association between the endocrine phenotype and stage (13). The above symptoms may be considered as a warning sign of adrenocortical carcinomas and can be further studied as prognosis indicators.

Currently, the preferred criteria for the diagnosis of pediatric adrenocortical carcinoma are those described by Wieneke et al (45). It mainly includes the following 9 criteria: tumor weight > 400 g; tumor diameter > 10.5 cm; Extension into the periadrenal soft tissue or adjacent organs; invasion into vena cava; vascular invasion; capsular invasion; the presence of tumor necrosis; mitoses > 15 per 20 high-power fields (4 mm2); and presence of atypical mitosis. Adrenocortical carcinoma can be diagnosed if 4 or more of the 9 items are satisfied. If less than or equal to 2 items, it is diagnosed as benign, and if any 3 items are satisfied, it is diagnosed as uncertain malignant potential. In addition, Ki67 has been proposed as an auxiliary biomarker to differentiate childhood adrenal adenoma from adrenal carcinoma and to predict tumor behavior. A Ki67 index of less than 10% is associated with benign disease, and a marker index of greater than 15% is associated with a higher risk of malignancy or adverse outcomes. The p53 gene is an important tumor suppressor gene in the human body. Its wild type makes cancer cells apoptotic, thereby preventing canceration. It has the function of helping cells to repair defects in genes (5, 46). The mutant type of p53 will increase canceration. Multiple studies have found that p53 is overexpressed in adrenocortical carcinomas, whereas it is normal in adrenal adenomas. Adrenocortical carcinomas are present in excess among carriers of germline p53 mutations, and, p53-associated Adrenocortical carcinomas occur predominantly in the pediatric age group (47). Unfortunately, the specific diagnostic criteria, Ki67 index, and p53 mutation of adrenocortical carcinoma could not be provided in our study. This is a limitation of this study that needs to be pointed out. However, the pediatric adrenocortical carcinoma patients included in our study were all pathologically diagnosed under rigorous scrutiny.

Like neuroblastoma, the treatment of adrenocortical carcinoma is still a surgery-based comprehensive treatment. Complete tumor resection to achieve negative margins is an important prognostic factor (8). Stage I patients are curable with surgery alone (48). Retroperitoneal lymph node dissection has failed to improve outcomes in patients with larger tumors (stage II), and its role as an independent treatment strategy is uncertain (13). Combination of surgery and chemotherapy shows good outcomes in patients with stage III adrenocortical carcinoma, but mitotane- and cisplatin-based regimens lead to higher toxicity in patients with metastatic disease, and efficacy in patients with metastatic disease was still poor and should be revised to maximize risk-benefit (13).

Drug therapy is often required to control tumor growth and excessive hormone secretion for patients who cannot achieve complete tumor resection and for patients with recurrence. Mitotane is currently the most effective drug to control adrenal hormone secretion, and it is used alternately with steroid hormones during use. It can be used as a single drug for adjuvant therapy after complete resection of an early tumor, or it can be used in combination with chemotherapy drugs for advanced childhood adrenal cortical carcinoma (13). Mitotane combined with chemotherapy improves the prognosis of patients with advanced adrenocortical carcinoma. However, not all patients can bear the drug’s side effects and can complete all the courses of the treatment as prescribed due to its severe toxicity and side effects (8, 13, 49). The common toxicities of mitotane are nausea, vomiting, diarrhea, and abdominal pain. Drowsiness, lethargy, ataxic gait, depression, and vertigo have also been reported in a few cases. Common chemotherapeutic agents used alone or in combination to treat adrenocortical carcinoma include 5-FU, etoposide, cisplatin, carboplatin, cyclophosphamide, doxorubicin, and streptozotocin. Chemotherapy regimens used to treat childhood adrenocortical carcinoma consist primarily of etoposide and cisplatin, with or without doxorubicin and mitotane. Despite the diverse treatment modalities of chemotherapy, the prognosis for advanced cases remains poor (8). Unfortunately, new therapeutic strategies targeting tumor-specific aberrant pathways have not been studied in pediatric adrenocortical carcinoma. And Radiation therapy in pediatric adrenocortical carcinoma needs further exploration (50).

According to The Children’s Oncology Group ARAR0332 Protocol (13), which is a prospective single-arm risk-stratified interventional study to describe the outcome of stage III or IV pediatric adrenocortical carcinoma patients treated with mitotane and chemotherapy. In the study, eight cycles of chemotherapy, and mitotane for 8 months were used for stages III and IV treatment; at the same time, according to clinical practice, surgical treatment of primary tumors and metastases may be considered as appropriate. The chemotherapy regimen was cisplatin 50 mg/m^2^/dose for 1-2 days, etoposide 100 mg/m^2^/dose for 1-3 days, and doxorubicin 25 mg/m^2^/dose for 4-5 days per cycle. Filgrastim was administered daily at 5 mcg/kg/dose starting on day 6 until neutrophil recovery. Treatment with mitotane is administered daily to adjust the plasma concentration of mitotane to 14-20 μg/mL. During the treatment, nearly one-third of patients were unable to complete their scheduled treatment. Finally, 38 patients have evaluated for toxicity or feasibility analysis and found that 4 patients had mitotane feasibility event (10.5%), and 12 patients had chemotherapy feasibility event (31.6%). It can be seen that it is necessary to modify the treatment plan to improve the tolerability of the treatment. What’s more, combining surgery and chemotherapy has a good prognosis in stage III patients and a poor prognosis in stage IV patients; with a median follow-up for OS of 60 months, the 5-year OS estimates for stages III, and IV were 94.7%, and 15.6%, respectively.

Note that mitotane produces multiple severe side effects on the metabolic and endocrine systems in the treatment of adrenocortical carcinoma, although this effect appears to be treatable and partially reversible. One study analyzed lipid profiles, thyroid hormones, sex hormones, and adrenal function in 50 patients from the first year of mitotane treatment and after discontinuation (51). In their study, they found levels of total cholesterol, LDL, HDL, and triglycerides increased after 6 months of mitotane treatment, and total cholesterol and LDL levels were reduced when statins were given concomitantly, and mitotane was discontinued Lipid can be further reduced; at the same time, it was also found that plasma free thyroxine decreased in the mitotane treatment group, but thyroid-stimulating hormone remained unchanged. The total amount of T4 increased when mitotane was discontinued. Mitotane increases plasma sex hormone-binding globulin and luteinizing hormone and increases the level of testosterone in male patients; in addition, the adrenal function can be recovered after six months of discontinuation of mitotane. Therefore, during the treatment of adrenocortical carcinoma with mitotane, in addition to causing adrenal insufficiency, special attention should be paid to the interference of mitotane on lipid metabolism and endocrine, and it should be corrected to normal levels in time. Patients taking mitotane may require high-dose hydrocortisone replacement therapy, and reducing mitotane can interfere with steroid metabolism (52).

Because of the heterogeneity and rarity of pediatric adrenocortical carcinoma, the short follow-up time, and the fact that most patients are diagnosed at an advanced stage of the disease, it is often challenging to identify prognostic factors. Multiple studies have found that age and tumor stage are two independent predictors (4, 13, 53, 54). Other risk factors include tumor size/tumor volume/weight, surgical treatment, presence of virilization, Cushing syndrome, and hypertension. Germline TP53 status and the presence of a somatic ATRX mutation were also associated with the outcome (8, 13).

This study was retrospective and had a few limitations. Firstly, the research data comes from the SEER database, which has not recorded specific radiotherapy, chemotherapy, or targeted treatment plans and lacks laboratory test results (such as NSE, VMA, LDH, cortisol, etc.) and medical data (mitotane dosage, side effects, need for substitutive treatment with cortisone, fludrocortisone and/or levothyroxine). Additionally, due to missing values, there may be some selection bias in the process of screening cases in the study.

## Conclusion

This study retrospectively performed a prognostic analysis of pediatric adrenal malignancies using a large sample size. Neuroblastoma, ganglioneuroblastoma, and adrenocortical carcinoma were the three most common pediatric adrenal malignancies. Patients with neuroblastoma and ganglioneuroblastoma often accompany metastatic lesions at presentation, making treatment challenging. Complete surgical resection of the tumor is the key to ensuring a good prognosis. Radiation and chemotherapy should be given to patients whose tumors cannot be entirely removed by surgery. The prognosis of pediatric adrenal malignancies is related to the age of the patient (older children have a poorer prognosis), type of the pathology (cortical cancer has a poor prognosis), tumor size (the larger the tumor, the worse the prognosis), and the tumor stage (the higher the stage of the patient). Notably, our study demonstrated that chemotherapy patients had a worse prognosis than those who did not. This was mainly related to the fact that patients receiving chemotherapy tend to reside in non-metropolitan regions, the pathological type was neuroblastoma, the tumor diameter was larger, the stage was higher, and the patients had not received surgery. Although the field of oncology have progressed with respect to treatment of pediatric adrenal malignancies, there still no statistically significant reduction in the risk of all-cause mortality and tumor-specific mortality in patients with recently diagnosed pediatric adrenal malignancies compared with patients in the past diagnosis period. Therefore, more experimental studies are needed in this regard urgently and efficiently to save lives

## Data availability statement

The original contributions presented in the study are included in the article/**Supplementary Material**. Further inquiries can be directed to the corresponding authors.

## Author contributions

ZL, XM, and SC conceived the research. ZL wrote the manuscript. ZL, YY, and YL analyzed the data and prepared the figures and tables. All authors contributed to the article and approved the submitted version.

## Acknowledgments

We thank the SEER database supported by the Surveillance Research Program in the National Cancer Institute’s Division of Cancer Control and Population Sciences. And we thank Bullet Edits Limited for the linguistic editing and proofreading of the manuscript.

## Conflict of interest

The authors declare that the research was conducted in the absence of any commercial or financial relationships that could be construed as a potential conflict of interest.

## Publisher’s note

All claims expressed in this article are solely those of the authors and do not necessarily represent those of their affiliated organizations, or those of the publisher, the editors and the reviewers. Any product that may be evaluated in this article, or claim that may be made by its manufacturer, is not guaranteed or endorsed by the publisher.

## References

[1] Xu X Sergi C. Pediatric adrenal cortical carcinomas: Histopathological criteria and clinical trials. A systematic review Contemp Clin Trials (2016) 50:37–44. doi: 10.1016/j.cct.2016.07.011 27424218

[2] Lin X Wu D Chen C Zheng N. Clinical characteristics of adrenal tumors in children: a retrospective review of a 15-year single-center experience. Int Urol Nephrol (2017) 49(3):381–5. doi: 10.1007/s11255-016-1480-z 27988912

[3] Abib SCV Weldon CB. Management of adrenal tumors in pediatric patients. Surg Oncol Clin N Am (2021) 30(2):275–90. doi: 10.1016/j.soc.2020.11.012 33706900

[4] Miele E Di Giannatale A Crocoli A Cozza R Serra A Castellano A. Clinical, genetic, and prognostic features of adrenocortical tumors in children: A 10-year single-center experience. Front Oncol (2020) 10:554388. doi: 10.3389/fonc.2020.554388 33178583 PMC7593337

[5] Mete O Erickson LA Juhlin CC de Krijger RR Sasano H Volante M. Overview of the 2022 WHO classification of adrenal cortical tumors. Endocr Pathol (2022) 33(1):155–96. doi: 10.1007/s12022-022-09710-8 PMC8920443 35288842

[6] Özcan HN Tan AA Ardıçlı B Oguz B Ekinci S Kutluk T. Imaging findings of primary adrenal tumors in pediatric patients. Diagn Interv Radiol (2021) 27(6):811–5. doi: 10.5152/dir.2021.20701 PMC8621640 34792039

[7] Croteau N Nuchtern J LaQuaglia MP. Management of neuroblastoma in pediatric patients. Surg Oncol Clin N Am (2021) 30(2):291–304. doi: 10.1016/j.soc.2020.11.010 33706901

[8] Pinto EM Zambetti GP Rodriguez-Galindo C. Pediatric adrenocortical tumours. Best Pract Res Clin Endocrinol Metab (2020) 34(3):101448. doi: 10.1016/j.beem.2020.101448 32636100

[9] Erdmann F Frederiksen LE Bonaventure A Mader L Hasle H Robison LL. Childhood cancer: Survival, treatment modalities, late effects and improvements over time. Cancer Epidemiol (2021) 71(Pt B). doi: 10.1016/j.canep.2020.101733 32461035

[10] Winther JF Kenborg L Byrne J Hjorth L Kaatsch P Kremer LC. Childhood cancer survivor cohorts in Europe. Acta Oncol (2015) 54(5):655–68. doi: 10.3109/0284186x.2015.1008648 25813473

[11] Yao W Dong K Li K Zheng S Xiao X. Comparison of long-term prognosis of laparoscopic and open adrenalectomy for local adrenal neuroblastoma in children. Pediatr Surg Int (2018) 34(8):851–6. doi: 10.1007/s00383-018-4294-5 29881893

[12] Kelleher CM Smithson L Nguyen LL Casadiego G Nasr A Irwin MS. Clinical outcomes in children with adrenal neuroblastoma undergoing open versus laparoscopic adrenalectomy. J Pediatr Surg (2013) 48(8):1727–32. doi: 10.1016/j.jpedsurg.2013.03.056 23932613

[13] Rodriguez-Galindo C Krailo MD Pinto EM Pashankar F Weldon CB Huang L. Treatment of pediatric adrenocortical carcinoma with surgery, retroperitoneal lymph node dissection, and chemotherapy: The children's oncology group ARAR0332 protocol. J Clin Oncol (2021) 39(22):2463–73. doi: 10.1200/jco.20.02871 PMC8462560 33822640

[14] Fascetti-Leon F Scotton G Pio L Beltrà R Caione P Esposito C. Minimally invasive resection of adrenal masses in infants and children: results of a European multi-center survey. Surg Endosc (2017) 31(11):4505–12. doi: 10.1007/s00464-017-5506-0 28550366

[15] Mattone MC Gil S Costanzo M Galluzzo Mutti ML Casanovas A Zaidman V. Pediatric adrenocortical tumors cohort characteristics and long-term follow-up at a single Argentinian tertiary center. J Pediatr Endocrinol Metab (2022) 35(1):19–27. doi: 10.1515/jpem-2021-0392 34674406

[16] Dong R Yang R Zhan Y Lai HD Ye CJ Yao XY. Single-cell characterization of malignant phenotypes and developmental trajectories of adrenal neuroblastoma. Cancer Cell (2020) 38(5):716–33.e6. doi: 10.1016/j.ccell.2020.08.014 32946775

[17] Swift CC Eklund MJ Kraveka JM Alazraki AL. Updates in diagnosis, management, and treatment of neuroblastoma. Radiographics (2018) 38(2):566–80. doi: 10.1148/rg.2018170132 29528815

[18] Duckett JW Koop CE. Neuroblastoma. Urol Clin North Am (1977) 4(2):285–95. 331618

[19] Tsubota S Kadomatsu K. Origin and initiation mechanisms of neuroblastoma. Cell Tissue Res (2018) 372(2):211–21. doi: 10.1007/s00441-018-2796-z 29445860

[20] Schwab M. Amplification of n-myc as a prognostic marker for patients with neuroblastoma. Semin Cancer Biol (1993) 4(1):13–8. 8448374

[21] Matthay KK Maris JM Schleiermacher G Nakagawara A Mackall CL Diller L. Neuroblastoma. Nat Rev Dis Primers (2016) 2:16078. doi: 10.1038/nrdp.2016.78 27830764

[22] Lampis S Raieli S Montemurro L Bartolucci D Amadesi C Bortolotti S. The MYCN inhibitor BGA002 restores the retinoic acid response leading to differentiation or apoptosis by the mTOR block in MYCN-amplified neuroblastoma. J Exp Clin Cancer Res (2022) 41(1):160. doi: 10.1186/s13046-022-02367-5 35490242 PMC9055702

[23] Peinemann F van Dalen EC Enk H Berthold F. Retinoic acid postconsolidation therapy for high-risk neuroblastoma patients treated with autologous haematopoietic stem cell transplantation. Cochrane Database Syst Rev (2017) 8(8):Cd010685. doi: 10.1002/14651858.CD010685.pub3 28840597 PMC6483698

[24] Pugh TJ Morozova O Attiyeh EF Asgharzadeh S Wei JS Auclair D. The genetic landscape of high-risk neuroblastoma. Nat Genet (2013) 45(3):279–84. doi: 10.1038/ng.2529 PMC3682833 23334666

[25] Attiyeh EF London WB Mossé YP Wang Q Winter C Khazi D. Chromosome 1p and 11q deletions and outcome in neuroblastoma. N Engl J Med (2005) 353(21):2243–53. doi: 10.1056/NEJMoa052399 16306521

[26] Gilbert F Feder M Balaban G Brangman D Lurie DK Podolsky R. Human neuroblastomas and abnormalities of chromosomes 1 and 17. Cancer Res (1984) 44(11):5444–9. 6488196

[27] Mlakar V Jurkovic Mlakar S Lopez G Maris JM Ansari M Gumy-Pause F. 11q deletion in neuroblastoma: a review of biological and clinical implications. Mol Cancer (2017) 16(1):114. doi: 10.1186/s12943-017-0686-8 28662712 PMC5492892

[28] Spitz R Hero B Simon T Berthold F. Loss in chromosome 11q identifies tumors with increased risk for metastatic relapses in localized and 4S neuroblastoma. Clin Cancer Res (2006) 12(11 Pt 1):3368–73. doi: 10.1158/1078-0432.Ccr-05-2495 16740759

[29] Brodeur GM Pritchard J Berthold F Carlsen NL Castel V Castelberry RP. Revisions of the international criteria for neuroblastoma diagnosis, staging, and response to treatment. J Clin Oncol (1993) 11(8):1466–77. doi: 10.1200/jco.1993.11.8.1466 8336186

[30] Evans AE D'Angio GJ Propert K Anderson J Hann HW. Prognostic factor in neuroblastoma. Cancer (1987) 59(11):1853–9. doi: 10.1002/1097-0142(19870601)59:11<1853::aid-cncr2820591102>3.0.co;2-f 3567848

[31] Li J Liu X Chen M Wang J Wang X. Values of serum CA125, NSE and 24-hour urine VMA in diagnosis and prediction of treatment of paediatric neuroblastoma. Int J Clin Pract (2021) 75(12):e14932. doi: 10.1111/ijcp.14932 34606672

[32] Voglino V Persano G Crocoli A Castellano A Serra A Giordano U. Hemorrhage during induction chemotherapy in neuroblastoma: Additional risk factors in high-risk patients. Front Pediatr (2021) 9:761896. doi: 10.3389/fped.2021.761896 34869118 PMC8635199

[33] Moroz V Machin D Hero B Ladenstein R Berthold F Kao P. The prognostic strength of serum LDH and serum ferritin in children with neuroblastoma: A report from the international neuroblastoma risk group (INRG) project. Pediatr Blood Cancer (2020) 67(8):e28359. doi: 10.1002/pbc.28359 32472746

[34] Whittle SB Smith V Doherty E Zhao S McCarty S Zage PE. Overview and recent advances in the treatment of neuroblastoma. Expert Rev Anticancer Ther (2017) 17(4):369–86. doi: 10.1080/14737140.2017.1285230 28142287

[35] Laprie A Michon J Hartmann O Munzer C Leclair MD Coze C. High-dose chemotherapy followed by locoregional irradiation improves the outcome of patients with international neuroblastoma staging system stage II and III neuroblastoma with MYCN amplification. Cancer (2004) 101(5):1081–9. doi: 10.1002/cncr.20453 15329919

[36] Park JR Kreissman SG London WB Naranjo A Cohn SL Hogarty MD. Effect of tandem autologous stem cell transplant vs single transplant on event-free survival in patients with high-risk neuroblastoma: A randomized clinical trial. Jama (2019) 322(8):746–55. doi: 10.1001/jama.2019.11642 PMC6714031 31454045

[37] Du L Liu L Zhang C Cai W Wu Y Wang J. Role of surgery in the treatment of patients with high-risk neuroblastoma who have a poor response to induction chemotherapy. J Pediatr Surg (2014) 49(4):528–33. doi: 10.1016/j.jpedsurg.2013.11.061 24726106

[38] Ladenstein R Pötschger U Pearson ADJ Brock P Luksch R Castel V. Busulfan and melphalan versus carboplatin, etoposide, and melphalan as high-dose chemotherapy for high-risk neuroblastoma (HR-NBL1/SIOPEN): An international, randomised, multi-arm, open-label, phase 3 trial. Lancet Oncol (2017) 18(4):500–14. doi: 10.1016/s1470-2045(17)30070-0 28259608

[39] Alexander N Vali R Ahmadzadehfar H Shammas A Baruchel S. Review: The role of radiolabeled DOTA-conjugated peptides for imaging and treatment of childhood neuroblastoma. Curr Radiopharm (2018) 11(1):14–21. doi: 10.2174/1874471011666171215093112 29243585

[40] Anongpornjossakul Y Sriwatcharin W Thamnirat K Chamroonrat W Kositwattanarerk A Utamakul C. Iodine-131 metaiodobenzylguanidine (131I-mIBG) treatment in relapsed/refractory neuroblastoma. Nucl Med Commun (2020) 41(4):336–43. doi: 10.1097/mnm.0000000000001152 31939898

[41] Yu AL Gilman AL Ozkaynak MF London WB Kreissman SG Chen HX. Anti-GD2 antibody with GM-CSF, interleukin-2, and isotretinoin for neuroblastoma. N Engl J Med (2010) 363(14):1324–34. doi: 10.1056/NEJMoa0911123 PMC3086629 20879881

[42] Chudler RM Kay R. Adrenocortical carcinoma in children. Urol Clin North Am (1989) 16(3):469–79. 2665273

[43] Pinto EM Chen X Easton J Finkelstein D Liu Z Pounds S. Genomic landscape of paediatric adrenocortical tumours. Nat Commun (2015) 6:6302. doi: 10.1038/ncomms7302 25743702 PMC4352712

[44] Zheng S Cherniack AD Dewal N Moffitt RA Danilova L Murray BA. Comprehensive pan-genomic characterization of adrenocortical carcinoma. Cancer Cell (2016) 29(5):723–36. doi: 10.1016/j.ccell.2016.04.002 PMC4864952 27165744

[45] Wieneke JA Thompson LD Heffess CS. Adrenal cortical neoplasms in the pediatric population: a clinicopathologic and immunophenotypic analysis of 83 patients. Am J Surg Pathol (2003) 27(7):867–81. doi: 10.1097/00000478-200307000-00001 12826878

[46] Das S Sengupta M Islam N Roy P Datta C Mishra PK. Weineke criteria, ki-67 index and p53 status to study pediatric adrenocortical tumors: Is there a correlation? J Pediatr Surg (2016) 51(11):1795–800. doi: 10.1016/j.jpedsurg.2016.07.014 27567308

[47] Wasserman JD Zambetti GP Malkin D. Towards an understanding of the role of p53 in adrenocortical carcinogenesis. Mol Cell Endocrinol (2012) 351(1):101–10. doi: 10.1016/j.mce.2011.09.010 PMC3288384 21930187

[48] Ribeiro RC Pinto EM Zambetti GP Rodriguez-Galindo C. The international pediatric adrenocortical tumor registry initiative: contributions to clinical, biological, and treatment advances in pediatric adrenocortical tumors. Mol Cell Endocrinol (2012) 351(1):37–43. doi: 10.1016/j.mce.2011.10.015 22040600

[49] Zancanella P Pianovski MA Oliveira BH Ferman S Piovezan GC Lichtvan LL. Mitotane associated with cisplatin, etoposide, and doxorubicin in advanced childhood adrenocortical carcinoma: mitotane monitoring and tumor regression. J Pediatr Hematol Oncol (2006) 28(8):513–24. doi: 10.1097/01.mph.0000212965.52759.1c 16912591

[50] Wiegering V Riedmeier M Thompson LDR Virgone C Redlich A Kuhlen M. Radiotherapy for pediatric adrenocortical carcinoma - review of the literature. Clin Transl Radiat Oncol (2022) 35:56–63. doi: 10.1016/j.ctro.2022.05.003 35601796 PMC9121070

[51] Vikner ME Krogh J Daugaard G Andreassen M. Metabolic and hormonal side effects of mitotane treatment for adrenocortical carcinoma: A retrospective study in 50 Danish patients. Clin Endocrinol (Oxf) (2021) 94(2):141–9. doi: 10.1111/cen.14345 32996176

[52] Oddie PD Albert BB Hofman PL Jefferies C Laughton S Carter PJ. Mitotane in the treatment of childhood adrenocortical carcinoma: A potent endocrine disruptor. Endocrinol Diabetes Metab Case Rep (2018) 2018. doi: 10.1530/edm-18-0059 PMC6109212 30159150

[53] Evanoff JD Patel SG Hickey KJ Rensing AJ. Survival characteristics of localized pediatric adrenocortical carcinoma managed with adenectomy: A national cancer center database analysis. J Pediatr Urol (2021) 17(5):735. doi: 10.1016/j.jpurol.2021.06.005 34210620

[54] Gulack BC Rialon KL Englum BR Kim J Talbot LJ Adibe OO. Factors associated with survival in pediatric adrenocortical carcinoma: An analysis of the national cancer data base (NCDB). J Pediatr Surg (2016) 51(1):172–7. doi: 10.1016/j.jpedsurg.2015.10.039 PMC5131646 26572849

