# Supplementary material for: Long-term survival outcomes of pediatric adrenal malignancies: An analysis with the upstaged SEER registry during 2000-2019
Source: Front Endocrinol (Lausanne). 2022 Sep 12;13:977105. doi: 10.3389/fendo.2022.977105 (PMC9511147; doi:10.3389/fendo.2022.977105)
Supplement: Supplementary file 1 [file Table_1.pdf]

Supplementary Table 1. Baseline characteristics of study cohort by chemotherapy and association factors of patients treated with chemotherapy.

	**Chemotherapy**			**Univariable**		**Multivariable (full model**		**Multivariable (reduced model**	
	**No/Unknown N=358 (22.4%)**	**Yes N=1243 (17.6%)**	**P**	**OR (95%CI)**	**P**	**OR (95%CI)**	**P**	**OR (95%CI)**	P
**Year at diagnosis**			0.008						
2000-2004	86 (24.0%)	288 (23.2%)		1 reference		1 reference		1 reference	
2005-2009	117 (32.7%)	304 (24.5%)		0.78 (0.56-1.07)	0.122	1.14 (0.69-1.87)	0.607	1.19 (0.73-1.94)	0.49
2010-2014	81 (22.6%)	330 (26.5%)		1.22 (0.86-1.71)	0.262	1.61 (0.96-2.71)	0.07	1.69 (1.01-2.81)	0.044
2015-2019	74 (20.7%)	321 (25.8%)		1.30 (0.91-1.84)	0.146	2.01 (1.17-3.46)	0.012	2.00 (1.17-3.44)	0.012
**Age at diagnosis**			<0.001						
0-4 year	273 (76.3%)	1006 (80.9%)		1 reference		1 reference		1 reference	
5-9 year	38 (10.6%)	152 (12.2%)		1.09 (0.74-1.59)	0.672	1.63 (0.88-2.99)	0.118	1.56 (0.85-2.85)	0.149
10-14 year	21 (5.87%)	52 (4.18%)		0.67 (0.40-1.13)	0.137	1.20 (0.46-3.08)	0.712	1.15 (0.45-2.95)	0.764
15+ year	26 (7.26%)	33 (2.65%)		0.34 (0.20-0.59)	<0.001	0.32 (0.12-0.82)	0.018	0.31 (0.12-0.79)	0.014
**Sex**			0.146						
Female	174 (48.6%)	548 (44.1%)		1 reference		1 reference			
Male	184 (51.4%)	695 (55.9%)		1.20 (0.95-1.52)	0.13	0.96 (0.67-1.37)	0.812		
**Race**			0.033						
White	294 (82.1%)	940 (75.6%)		1 reference		1 reference			
Black	39 (10.9%)	175 (14.1%)		1.40 (0.97-2.03)	0.073	1.28 (0.72-2.28)	0.396		
Other	25 (6.98%)	128 (10.3%)		1.60 (1.02-2.51)	0.04	1.35 (0.69-2.66)	0.38		
**Median household income**			0.759						
$0-$59999	91 (25.4%)	315 (25.3%)		1 reference		1 reference			
$60000-$69999	106 (29.6%)	392 (31.5%)		1.07 (0.78-1.47)	0.683	0.69 (0.40-1.17)	0.17		
$70000+	161 (45.0%)	536 (43.1%)		0.96 (0.72-1.29)	0.794	0.74 (0.44-1.24)	0.254		
**Residence**			0.021						
Metropolitan	312 (87.2%)	1136 (91.4%)		1 reference		1 reference		1 reference	

Nonmetropolitan	46 (12.8%)	107 (8.61%)		0.64 (0.44-0.92)	0.017	0.40 (0.21-0.77)	0.006	0.49 (0.28-0.86)	0.013
**Histology**			<0.001						
Adrenal cortical carcinoma	37 (10.3%)	52 (4.18%)		1 reference		1 reference		1 reference	
Ganglioneuroblastoma	70 (19.6%)	81 (6.52%)		0.82 (0.49-1.40)	0.472	0.75 (0.32-1.77)	0.516	0.79 (0.34-1.86)	0.59
Neuroblastoma	235 (65.6%)	1100 (88.5%)		3.33 (2.14-5.19)	<0.001	1.56 (0.73-3.36)	0.253	1.59 (0.74-3.42)	0.239
Other	16 (4.47%)	10 (0.80%)		0.44 (0.18-1.09)	0.076	0.25 (0.06-1.07)	0.061	0.25 (0.06-1.06)	0.059
**Size. per cm**	53.0 [35.0;72.0]	81.0 [53.0;110]	<0.001	1.07 (1.04-1.09)	<0.001	1.10 (1.06-1.15)	<0.001	1.10 (1.06-1.15)	<0.001
**Stage**			<0.001						
Distant	52 (14.5%)	1057 (85.0%)		1 reference		1 reference		1 reference	
Localized	225 (62.8%)	45 (3.62%)		0.01 (0.01-0.02)	<0.001	0.01 (0.01-0.02)	<0.001	0.01 (0.01-0.02)	<0.001
Regional	81 (22.6%)	141 (11.3%)		0.09 (0.06-0.13)	<0.001	0.08 (0.05-0.12)	<0.001	0.08 (0.05-0.12)	<0.001
**Surgical treatment**			<0.001						
No	12 (3.35%)	280 (22.5%)		1 reference		1 reference		1 reference	
Local tumor destruction/excision	52 (14.5%)	87 (7.00%)		0.07 (0.04-0.14)	<0.001	0.11 (0.05-0.27)	<0.001	0.12 (0.05-0.27)	<0.001
Radical surgery with or not other organs	209 (58.4%)	672 (54.1%)		0.14 (0.08-0.25)	<0.001	0.23 (0.11-0.48)	<0.001	0.23 (0.11-0.46)	<0.001
Simple/partial surgical removal	85 (23.7%)	204 (16.4%)		0.10 (0.05-0.19)	<0.001	0.20 (0.09-0.43)	<0.001	0.19 (0.09-0.42)	<0.001
**Surgery for non-primary other distant sites**			<0.001						
None	334 (93.3%)	1059 (85.2%)		1 reference		1 reference			
Yes	24 (6.70%)	184 (14.8%)		2.42 (1.55-3.76)	<0.001	0.80 (0.45-1.42)	0.451		
**Months to treatment**			<0.001						
0 months	285 (79.6%)	937 (75.4%)		1 reference		1 reference		1 reference	
1 months	50 (14.0%)	281 (22.6%)		1.71 (1.23-2.37)	0.001	0.95 (0.59-1.52)	0.817	0.99 (0.61-1.58)	0.951
2+ months	23 (6.42%)	25 (2.01%)		0.33 (0.18-0.59)	<0.001	0.23 (0.10-0.56)	0.001	0.23 (0.10-0.56)	0.001

IQR: Interquartile range; OR: odds ratio; CI: Confidence interval.

Supplementary Table 2. Five-year overall survival (OS) and cancer-special survival (CSS) and predictors of all-cause mortality for patients with adrenal neuroblastoma and ganglioneuroblastoma.

	5-OS (95%CI)	5-CSS (95%CI)	Univariable		Multivariable	
			HR (95%CI)	P	HR (95%CI)	P
**Year at diagnosis**						
2000-2004	69.0% [64.3%-74.0%]	69.5% [64.8%-74.5%]	1 reference		1 reference	
2005-2009	69.4% [65.0%-74.1%]	70.4% [66.0%-75.1%]	1.04 [0.81-1.32]	0.774	1.11 [0.87-1.42]	0.4156
2010-2014	71.7% [67.2%-76.5%]	73.1% [68.7%-77.9%]	0.90 [0.69-1.16]	0.414	0.83 [0.63-1.10]	0.1955
2015-2019 *	78.2% [72.8%-83.9%]	79.0% [73.6%-84.7%]	0.81 [0.59-1.13]	0.217	0.78 [0.55-1.09]	0.1418
**Age at diagnosis**						
0-4 year	72.4% [69.7%-75.1%]	73.5% [70.9%-76.2%]	1 reference		1 reference	
5-9 year	61.5% [54.0%-70.1%]	62.4% [54.9%-71.0%]	1.49 [1.14-1.94]	0.003	1.39 [1.06-1.81]	0.0175
10-14 year	63.8% [50.9%-79.8%]	63.8% [50.9%-79.8%]	1.68 [1.08-2.61]	0.022	1.66 [1.04-2.65]	0.0346
15+ year	55.9% [40.9%-76.4%]	55.9% [40.9%-76.4%]	1.97 [1.21-3.21]	0.006	2.16 [1.15-4.08]	0.0172
**Sex**						
Female	70.6% [66.9%-74.5%]	71.7% [68.1%-75.6%]	1 reference		1 reference	
Male	70.3% [67.1%-73.7%]	71.3% [68.0%-74.7%]	1.07 [0.88-1.29]	0.512	0.97 [0.80-1.18]	0.7947
**Race**						
White	73.1% [70.4%-76.0%]	73.7% [71.0%-76.5%]	1 reference		1 reference	
Black	63.0% [56.2%-70.6%]	64.9% [58.1%-72.4%]	1.45 [1.12-1.86]	0.004	1.31 [1.01-1.70]	0.0394
Other	60.3% [52.3%-69.6%]	63.3% [55.2%-72.5%]	1.59 [1.18-2.13]	0.002	1.46 [1.08-1.97]	0.0137
**Median household income**						
$0-$59999	65.5% [60.5%-70.9%]	67.1% [62.2%-72.4%]	1 reference		1 reference	
$60000-$69999	70.3% [65.9%-74.9%]	71.2% [66.9%-75.9%]	0.84 [0.66-1.08]	0.172	0.90 [0.69-1.18]	0.4468
$70000+	73.6% [70.0%-77.4%]	74.3% [70.8%-78.1%]	0.75 [0.60-0.95]	0.015	0.84 [0.65-1.10]	0.2061
**Residence**						
Metropolitan	71.7% [69.1%-74.3%]	72.7% [70.1%-75.3%]	1 reference		1 reference	
Nonmetropolitan	58.9% [50.6%-68.5%]	60.0% [51.7%-69.6%]	1.52 [1.15-2.02]	0.004	1.34 [0.97-1.86]	0.0788

**Histology**						
Ganglioneuroblastoma	76.8% [69.6%-84.8%]	76.8% [69.6%-84.8%]	1 reference		1 reference	
Neuroblastoma	69.9% [67.3%-72.7%]	71.1% [68.5%-73.8%]	1.19 [0.85-1.67]	0.314	0.83 [0.59-1.17]	0.2902
Other	62.3% [45.4%-85.3%]	62.3% [45.4%-85.3%]	1.83 [0.91-3.67]	0.091	2.05 [0.87-4.86]	0.1016
**Stage**						
Distant	60.7% [57.6%-64.0%]	61.9% [58.8%-65.2%]	1 reference		1 reference	
Localized	96.9% [94.5%-99.4%]	97.4% [95.2%-99.7%]	0.08 [0.04-0.16]	<0.001	0.15 [0.07-0.32]	<0.001
Regional	92.0% [88.0%-96.1%]	92.0% [88.0%-96.1%]	0.18 [0.12-0.30]	<0.001	0.22 [0.14-0.36]	<0.001
**Surgical treatment**						
No	63.7% [58.0%-70.1%]	65.3% [59.6%-71.6%]	1 reference		1 reference	
Local tumor destruction/excision	75.9% [68.3%-84.3%]	75.9% [68.3%-84.3%]	0.56 [0.37-0.84]	0.005	0.81 [0.52-1.25]	0.3354
Radical surgery with or not other organs	70.3% [67.0%-73.8%]	71.4% [68.1%-74.8%]	0.74 [0.59-0.94]	0.014	0.86 [0.66-1.12]	0.2681
Simple/partial surgical removal	75.3% [69.9%-81.2%]	76.2% [70.8%-82.0%]	0.57 [0.41-0.78]	0.001	0.70 [0.49-1.00]	0.0508
**Surgery for non-primary other distant site**						
None	72.0% [69.4%-74.7%]	73.0% [70.4%-75.6%]	1 reference		1 reference	
Yes	60.5% [53.4%-68.4%]	61.6% [54.6%-69.6%]	1.47 [1.15-1.90]	0.003	1.19 [0.92-1.54]	0.1778
**Radiotherapy**						
No	75.4% [72.7%-78.3%]	76.5% [73.8%-79.4%]	1 reference		1 reference	
Yes	60.0% [55.4%-65.0%]	60.9% [56.3%-65.9%]	1.57 [1.30-1.91]	<0.001	1.05 [0.84-1.31]	0.6858
**Chemotherapy**						
No/Unknown	94.7% [92.1%-97.4%]	95.4% [93.0%-97.9%]	1 reference		1 reference	
Yes	64.0% [61.1%-67.1%]	65.1% [62.2%-68.2%]	6.69 [4.27-10.48]	<0.001	2.36 [1.36-4.11]	0.0023
**Months to treatment**						
0 months	71.7% [68.9%-74.6%]	72.5% [69.7%-75.4%]	1 reference		1 reference	
1 months	64.5% [58.9%-70.5%]	66.3% [60.8%-72.3%]	1.30 [1.05-1.63]	0.018	1.04 [0.83-1.30]	0.7389
2+ months	80.7% [69.4%-93.8%]	80.7% [69.4%-93.8%]	0.82 [0.45-1.49]	0.515	1.23 [0.67-2.27]	0.5064

HR: hazard ratio; CI: confidence interval;

# Cox proportional risk regression model;

* 59 months survival rate.

Supplementary Table 3. Five-year overall survival (OS) and cancer-special survival (CSS) and predictors of all-cause mortality for patients with adrenal cortical carcinoma.

	5-OS (95%CI)	5-CSS (95%CI)	Univariable		Multivariable	
			HR (95%CI)	P	HR (95%CI)	P
**Year at diagnosis**						
2000-2004	57.1% [39.5%-82.8%]	57.1% [39.5%-82.8%]	1 reference		1 reference	
2005-2009	56.1% [37.4%-84.4%]	56.1% [37.4%-84.4%]	0.88 [0.35-2.23]	0.786	1.00 [0.29-3.49]	0.9981
2010-2014	55.0% [39.5%-76.5%]	55.0% [39.5%-76.5%]	0.88 [0.38-2.02]	0.764	0.55 [0.17-1.81]	0.3284
2015-2019 *	20.8% [4.3%-99.7%]	22.0% [4.6%-100.0%]	1.55 [0.62-3.89]	0.349	0.81 [0.22-3.02]	0.7505
**Age at diagnosis**						
0-4 year	83.5% [72.2%-96.5%]	83.5% [72.2%-96.5%]	1 reference		1 reference	
5-9 year	44.4% [21.4%-92.3%]	44.4% [21.4%-92.3%]	5.25 [1.69-16.32]	0.004	2.05 [0.40-10.42]	0.387
10-14 year	25.9% [11.4%-58.6%]	25.9% [11.4%-58.6%]	6.01 [2.26-15.95]	<0.001	1.75 [0.37-8.36]	0.4838
15+ year	19.8% [7.5%-52.6%]	20.9% [7.9%-55.2%]	8.03 [3.07-20.97]	<0.001	3.91 [0.92-16.64]	0.0648
**Sex**						
Female	52.2% [39.7%-68.7%]	53.3% [40.7%-69.9%]	1 reference		1 reference	
Male	52.5% [37.9%-72.7%]	52.5% [37.9%-72.7%]	0.92 [0.49-1.73]	0.797	0.94 [0.38-2.32]	0.8954
**Race**						
White	49.3% [38.8%-62.7%]	50.0% [39.4%-63.5%]	1 reference		1 reference	
Black	50.0% [22.5%-100.0%]	50.0% [22.5%-100.0%]	0.90 [0.28-2.93]	0.864	1.74 [0.29-10.42]	0.5439
**Median household income**						
$0-$59999	51.0% [33.3%-78.3%]	51.0% [33.3%-78.3%]				
$60000-$69999	53.2% [36.7%-77.0%]	53.2% [36.7%-77.0%]	0.91 [0.39-2.12]	0.834	1.10 [0.30-4.01]	0.8847
$70000+	52.9% [38.8%-72.1%]	54.4% [40.1%-73.8%]	1.00 [0.46-2.17]	0.998	0.48 [0.13-1.79]	0.276
**Residence**						
Metropolitan	49.2% [38.9%-62.3%]	49.9% [39.5%-63.1%]	1 reference		1 reference	
Nonmetropolitan	78.7% [56.4%-100.0%]	78.7% [56.4%-100.0%]	0.34 [0.08-1.42]	0.14	0.30 [0.05-1.96]	0.2088
**Stage**			1.03 [0.97-1.09]	0.382	1.00 [0.93-1.07]	0.9529
Distant	23.9% [13.5%-42.6%]	24.6% [13.9%-43.7%]				

Localized	90.3% [80.5%-100.0%]	90.3% [80.5%-100.0%]	0.07 [0.02-0.23]	<0.001	0.11 [0.02-0.54]	0.007
Regional	46.3% [24.5%-87.5%]	46.3% [24.5%-87.5%]	0.46 [0.19-1.12]	0.086	0.68 [0.20-2.36]	0.548
**Surgical treatment**						
No	8.5% [1.3%-54.8%]	9.6% [1.5%-60.7%]	1 reference		1 reference	
Radical surgery with or not other organs	51.6% [38.4%-69.6%]	51.7% [38.4%-69.6%]	0.19 [0.09-0.39]	<0.001	0.20 [0.06-0.66]	0.0084
Simple/partial surgical removal	71.4% [53.1%-96.2%]	71.4% [53.1%-96.2%]	0.10 [0.03-0.28]	<0.001	0.20 [0.04-1.02]	0.0526
**Surgery for non-primary other distant site**						
None	55.6% [45.1%-68.5%]	55.6% [45.1%-68.5%]	1 reference		1 reference	
Yes	30.0% [11.6%-77.3%]	33.8% [13.5%-84.5%]	1.94 [0.86-4.39]	0.111	1.42 [0.34-5.95]	0.6341
**Radiotherapy**						
No	53.5% [43.2%-66.2%]	54.2% [43.8%-67.0%]	1 reference		1 reference	
Yes	42.9% [18.2%-100.0%]	42.9% [18.2%-100.0%]	1.09 [0.39-3.08]	0.866	0.51 [0.08-3.36]	0.4803
**Chemotherapy**						
No/Unknown	79.8% [67.5%-94.5%]	82.2% [70.1%-96.3%]	1 reference		1 reference	
Yes	32.6% [21.5%-49.5%]	32.6% [21.5%-49.5%]	3.94 [1.80-8.60]	0.001	0.85 [0.27-2.66]	0.7808
**Months to treatment**						
0 months	52.7% [42.0%-66.0%]	53.4% [42.7%-66.8%]	1 reference		1 reference	
1 months	55.6% [32.8%-94.1%]	55.6% [32.8%-94.1%]	1.00 [0.39-2.55]	0.995	0.54 [0.17-1.71]	0.2967
2+ months	NA	NA	3.81 [0.51-28.50]	0.193	0.62 [0.05-7.30]	0.7029

HR: hazard ratio; CI: confidence interval;

# Cox proportional risk regression model;

* 59 months survival rate.
